# Performance and prognostic utility of the 92-gene assay in the molecular subclassification of ampullary adenocarcinoma

**DOI:** 10.1186/s12885-016-2677-3

**Published:** 2016-08-22

**Authors:** Michael J. Overman, Harris S. Soifer, Aaron Joel Schueneman, Joe Ensor, Volkan Adsay, Burcu Saka, Nastaran Neishaboori, Robert A. Wolff, Huamin Wang, Catherine A. Schnabel, Gauri Varadhachary

**Affiliations:** 1 Department of Gastrointestinal Medical Oncology, The University of Texas MD Anderson Cancer Center, Unit 426, 1515 Holcombe Blvd, Unit 421, Houston, TX 77030 USA; 2 Biotheranostics, Inc., 9620 Towne Centre Drive, Suite 200, San Diego, CA 92121 USA; 3 Houston Methodist Cancer Center, Houston Methodist Research Institute Methodist, Houston, TX USA; 4 Anatomic Pathology, Emory University School of Medicine, H180-B EUH, Atlanta, GA 30332 USA; 5 Department of Pathology, The University of Texas MD Anderson Cancer Center, 1515 Holcombe Blvd, Unit 085, Houston, TX 77030 USA

**Keywords:** Ampullary, Adenocarcinoma, Prognostic, Subclassification, 92-gene assay, Gene expression profiling, Biomarker

## Abstract

**Background:**

Ampullary adenocarcinoma is a rare gastrointestinal cancer associated with diverse outcomes due to clinical and pathological heterogeneity. Standardized methods to better prognosticate and inform therapeutic selection for ampullary adenocarcinoma are needed. This study explored the novel use and potential prognostic utility of a 92-gene cancer classifier in ampullary adenocarcinomas.

**Methods:**

In this prospectively-defined, blinded study of ampullary adenocarcinoma [N =54; stage T3 or higher (57 %); Grade III (44 %); Node positive (55 %)], the performance of a 92-gene classifier was examined to predict the ampullary subtype that was derived from histomorphological examination of resected ampullary samples. Outcome data for relapse-free survival (RFS) and overall survival (OS) were plotted to compare the prognostic utility of histological subtyping, histomolecular phenotyping, and the 92-gene classifier. Multivariate analysis was used to determine clinicopathological variables that were independently associated with overall survival.

**Results:**

The 92-gene classifier demonstrated sensitivities and specificities of 85 % [95 % CI, 66–94] and 68 % [95 % CI, 48–84] and 64 % [95 % CI, 46–79] and 88 % [95 % CI, 70–98] for the pancreaticobiliary and intestinal histological subtypes, respectively. For the 92-gene classifier, improved outcomes were observed for the intestine versus the pancreaticobiliary prediction (median OS 108.1 v 36.4 months; HR, 2.17; 95 % CI, 0.98 to 4.79; *P* = 0.05). Similar results were seen for ampullary adenocarcinoma stratification by histological subtype (*P* = 0.04) and histomolecular phenotype (*P* = 0.02). Within poorly differentiated ampullary adenocarcinomas only the 92-gene classifier demonstrated statistically significant differences in RFS and OS (*P* < 0.05).

**Conclusions:**

Prognostic stratification of ampullary adenocarcinoma was similar for the 92-gene classifier, histological subtype, and histomolecular phenotype. The 92-gene classifier provides an unbiased standardized molecular-based approach to stratify ampullary tumors.

**Electronic supplementary material:**

The online version of this article (doi:10.1186/s12885-016-2677-3) contains supplementary material, which is available to authorized users.

## Background

Adenocarcinomas of the ampulla of Vater are uncommon gastrointestinal cancers with heterogeneous outcomes. Histological classification of ampullary adenocarcinomas, and subsequent clinical management, is challenging due to the anatomical complexity and convergence of three distinct epithelial types in this region [1]. While previous studies suggest a role for histological classification of ampullary carcinoma into intestinal and pancreaticobiliary subtypes through examination of morphological features, the variability in subtype frequencies observed across studies has contributed to the unclear prognostic utility of histological examination alone [2–4]. Moreover, inter-pathologist concordance for subtyping ampullary adenocarcinomas is reportedly poor, with k value of 0.57 for intestinal and pancreaticobiliary subtypes, and a k value of 0.09 for the mixed subtype [5].

In addition to histological examination of morphological features, immunohistochemical (IHC) stains have been utilized to substantiate the intestinal versus pancreaticobiliary morphology of these adenocarcinomas including CK7, CK17, CK20 and CDX-2, and MUC1/2 [6]. Recently, Chang et al. described clinically relevant histomolecular phenotypes using both histomorphology and differential protein expression of CDX2 and MUC1 in patients with resected ampullary cancers [3]. While the histomolecular phenotype represents progress in classifying ampullary adenocarcinomas, IHC has challenges including tissue heterogeneity and antigenicity, interpretation of staining patterns, and inter/intra- observer variability [5, 7, 8]. Given the inherent subjective nature of both morphological evaluation and IHC, there exists a need for standardized approaches for tumor classification.

Genomic classifiers based on gene expression profiling of known reference tumor types have reported performance accuracies in the range of 80–90 % [9–13]. The 92-gene assay (CancerTYPE ID® Biotheranostics, Inc.) is a clinically validated cancer classifier that measures and interrogates the collective expression of 92 genes to determine tumor type and subtype utilizing a computational algorithm trained on a reference database of more than 2000 tumors [8, 13]. The objectives of the current study were to 1) evaluate the performance of the 92-gene classifier to subtype ampullary adenocarcinomas into intestine and pancreaticobiliary types, and 2) compare the prognostic utility of histological examination (morphology alone), histomolecular phenotype (morphology plus IHC) and the 92-gene classifier (genomic classification) to stratify ampullary adenocarcinomas into intestinal and pancreaticobiliary subtypes.

## Methods

### Study population and pathologic examination

Clinicopathological and outcome data for 79 patients with a diagnosis of ampullary adenocarcinoma who underwent a pancreaticoduodenectomy at the University of Texas, MD Anderson Cancer Center (UTMDACC) were included. The ampullary tumor site of origin was diagnosed based upon the original pancreaticoduodenectomy pathology report. Cases in which the ampullary tumor site of origin could not be ascertained were not included. Histological diagnosis (intestinal, pancreaticobiliary, or mixed) and pathological features for each case were reviewed by a specialist gastrointestinal pathologist who was blinded from clinical outcomes. Tumors were classified as intestinal (Int), pancreaticobiliary (Pb), or mixed [4]. Cases with mixed histology, containing more than 10 % of both histologic subtypes, were reclassified based upon the predominant histological subtype as either intestinal or pancreaticobiliary. Tumor staging was per the AJCC 7^th^ edition. Study approval was obtained from the Institutional Review Board at UTMDACC.

### Immunohistochemical scoring and histomolecular phenotype

Tissue microarrays were constructed as described previously [14] from formalin-fixed paraffin embedded (FFPE) material and consisted of three cores of tumor, and two cores of paired normal small bowel mucosa when available. Immunohistochemical staining was scored based on methods described by Chang et al. to maintain uniformity for comparisons [3]. For CDX2 (Biogenex, San Ramon, CA, USA; clone CDX-88), both the intensity and the percentage of positive cells were determined. Tumors with a modified H score > 35 were considered positive for CDX2 expression; MUC1 (Novocastra, Newcastle, UK; clone Ma695) positivity was defined as any positive staining [3]. Expression of CDX2 and MUC1 was combined with the histological subtype to classify ampullary adenocarcinomas into two different histomolecular phenotypes, pancreaticobiliary (Pb) and non-pancreaticobiliary (Non-Pb) as described [3]. Positive CK7 (Dako, Carpinteria, CA, USA; clone OVT-TL 12/30) and CK20 (Dako, Carpinteria, CA, USA; clone KS20.8) expression was defined as >10 % of tumor cells showing immunoreactivity.

### 92-gene classifier

For molecular profiling, 1 H&E and 3 unstained slides of FFPE tumor sections were submitted for each case in a blinded manner. Tumor cells were enriched by either macro-dissection H&E or laser microdissection. The 92-gene classifier (real-time RT-PCR) was performed on total RNA as previously described, and used a pre-specified computational algorithm that applies linear discriminant analysis to generate probabilities for candidate tumor types based on the degree of similarity of the queried sample to the reference tumor database [15]. Cases exceeding the PCR analytical cut-off for internal controls (PCR cycling threshold >30) were considered quality control (QC) failures. Cases were unblinded after testing was completed. The main cancer type prediction reported as the highest relative probability was utilized to evaluate assay performance.

### Statistical and survival analyses

Performance of the 92-gene classifier was evaluated based on concordance with the ampullary subtype established by histological (morphology alone) examination as the reference standard. Overall sensitivity (i.e. performance) was calculated as the number of cases with a main cancer type prediction that matched the reference histological subtype divided by the total number of cases classifiable by the assay. Sensitivity, specificity, positive predictive value (PPV) and negative predictive value (NPV) for each subtype were calculated as previously described [15]. For the 92-gene classifier, ROC curve and AUC analysis was performed using the rank order probability percentage of the main type cancer prediction.

Survival curves were generated using the Kaplan-Meier method. Survival differences were determined with the log-rank test. *P* values < 0.05 were considered statistically significant. Univariate Cox proportional hazards regression models were used for relapse-free survival (RFS) and overall survival (OS). Clinicopathological features with a *P* value < 0.05 were entered into multivariate Cox proportional hazard models for OS.

### 3-gene prognostic model

Cox regression models for OS and RFS was performed on each of the 87 informative genes separately to identify genes with a significant (*p*-value < 0.05) effect on outcome, either OS or RFS. For the 3-gene prognostic model, Cox regression analysis was performed using the expression levels of the three genes to calculate a linear combination of gene expression score for each sample. A linear combination score less than 0.45 was defined as improved prognosis, whereas a linear combination score greater than 0.45 was defined as poor prognosis [16]. Survival curves based on a cutpoint of 0.45 for the linear combination of gene expression were generated using the Kaplan-Meier method. Survival differences were determined with the log-rank test. *P* values < 0.05 were considered statistically significant.

## Results

### Study population

**Fig. 1:**
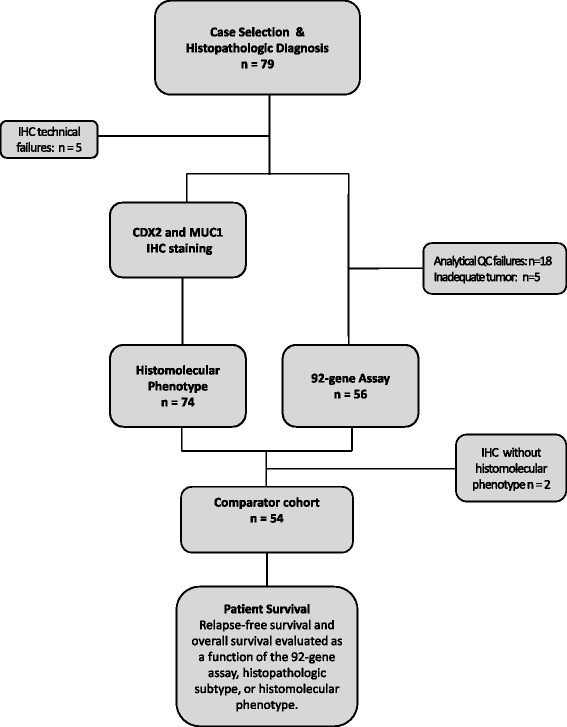
Case selection and flow diagram for molecular testing of ampullary adenocarcinoma. Seventy-nine cases of ampullary adenocarcinoma were identified for this study. Tumor specimens were assigned a reference histological subtype (intestinal or pancreaticobiliary) by a gastrointestinal pathologist. The comparator cohort comprised 54 cases to measure the prognostic performance of the 3 classifiers: 92-gene classifier, histological subtype, and histomolecular phenotype

Of 79 ampullary adenocarcinoma cases, 25 samples (32 %) did not pass quality control parameters; in a majority of these cases (*N* = 18), RNA quality was poor with attrition rates consistent with FFPE block ages > 10 years. Fifty-four tumor specimens were available to evaluate the performance of the 92-gene classifier to subtype ampullary adenocarcinomas (Fig. 1); an “off-panel” tumor type for which the classifier was initially untrained [15]. Patient demographic and clinicopathological features of the ampullary cohort are listed in Table 1. Clinicopathological features of the cohort associated with advanced disease include stage T2 disease or higher (88.7 %), high grade (44.4 %, Grade III alone) and regional lymph node metastasis (55.6 %). Histological (morphology alone) evaluation of the ampullary cohort characterized 20 tumors of intestinal subtype, 20 tumors of pancreaticobiliary subtype and 14 tumors of mixed histology. When only the predominant histology was considered, cases were classified as 28 intestinal and 26 pancreaticobiliary tumors (Table 1).

**Table 1:** Clinicopathologic features of ampullary adenocarcinomas

Clinicopathologic feature	No.	Percent	Median OS (months)	Log rank *P*
Sex				0.23
Male	35	64.8	103.8	
Female	19	35.2	245.2	
Age, years				0.16
Mean	64.6			
Median	66.5			
Range	34-83			
T stage^a^				0.15
T1	6	11.3	NA	
T2	17	32.1	103.8	
T3	21	39.6	39.7	
T4	9	17.0	108.1	
N stage				0.034
N0	24	44.4	113.8	
N1	30	55.6	41.8	
Grade				0.92
I	2	3.7	113.8	
II	28	51.9	63.7	
III	24	44.4	108.1	
Mucinous				0.17
Negative	50	92.6	108.1	
Positive + signet ring	4	7.4	45.6	
Perineural invasion				0.70
Negative	44	81.5	108.1	
Positive	10	18.5	94.8	
Lymphovascular invasion				0.46
Negative	38	70.4	113.8	
Positive	16	29.6	57.2	
Perioperative treatment				0.94
Neoadjuvant +/or adjuvant	25	46.3	145.9	
None	29	53.7	103.8	
Adenoma precursor lesion				0.56
Absent	33	61.1	113.8	
Present	21	38.9	63.7	
Resection margin				0.006
R0	52	96.3	108.1	
R1	2	3.7	18.8	
CK7 expression				0.70
Negative	12	22.2	63.7	
Positive	42	77.8	108.1	
CK20 expression				0.97
Negative	34	63.0	113.8	
Positive	20	37.0	103.8	
CDX2 expression^b^				0.93
Negative	33	61.1	108.1	
Positive	21	38.9	103.8	
MUC1 expression^c^				0.28
Negative	19	35.2	113.8	
Positive	35	64.8	63.72	
Histopathologic subtype^d^				0.035
Intestinal (plus 8 mixed histology)	28	52.0	108.1	
Pancreaticobiliary (plus 6 mixed histology)	26	48.0	34.6	
Histomolecular phenotype				0.017
Non-Pancreaticobiliary	40	74.1	113.8	
Pancreaticobiliary	14	25.9	28.0	
92-gene Classifier^e^				0.050
Intestinal	21	38.9	108.1	
Pancreaticobiliary	31	57.4	36.4	

*Abbreviations*: *OS*, overall survival

^a^T stage, N = 53, one case was carcinoma in situ

^b^Positive CDX2 expression was defined as modified H score > 35

^c^Positive MUC1 expression was defined as any positive staining

^d^ *P* value for the predominant subtype. The predominant subtypes for the 14 samples with mixed histology are also indicated

^e^Single gastroesophageal and single lung adenocarcinoma predictions excluded. *P* < 0.05 was considered statistically significant

### Performance of the 92-gene cancer classifier

The output from the 92-gene cancer classifier is a main cancer type prediction based on a comparison of the collective expression of 92-genes in the tumor sample with a database of reference tumors representing 28 main cancer types and over 50 cancer subtypes. Within this cohort, the main cancer type predictions by the 92-gene classifier were Pancreaticobiliary (*N* = 31; 57.4 %), Intestine (*N* = 21; 38.9 %), Gastroesophageal adenocarcinoma (*N* = 1; 1.9 %), and Lung adenocarcinoma (*N* = 1; 1.9 %) (Table 2). In addition to the main cancer type prediction, the 92-gene assay distinguishes between different subtypes of the main cancer type prediction as previously described [15]. Of the 21 ampullary adenocarcinomas classified by the 92-gene assay as intestinal, 14/21 (67 %) were subtyped as colorectal adenocarcinoma and 7/21 (33 %) were subtyped as small intestine adenocarcinoma. Of the 31 ampullary adenocarcinomas classified as pancreaticobiliary, 26/31 (84 %) were subtyped as gallbladder adenocarcinoma, 4/31 (13 %) were subtyped as cholangiocarcinoma, and 1/31 (3 %) were subtyped as pancreatic adenocarcinoma.

**Table 2:** Performance characteristics of the 92-gene classifier for the identification of histological subtypes in ampullary adenocarcinomas

Histopathologic category	N	Type of prediction (*N*)	Correct predictions	Sensitivity (95 % CI)	Specificity (95 % CI)	PPV	NPV
Ampullary-Intestinal	28	Intestine (18)	18	0.64 (0.46, 0.79)	0.88 (0.70, 0.98)	0.86	0.70
		Pancreaticobiliary (9)					
		Gastroesophageal (1)					
Ampullary-Pancreaticobiliary	26	Pancreaticobiliary (22)	22	0.85 (0.66, 0.94)	.68 (0.48, 0.84)	0.71	0.83
		Intestine (3)					
		Lung adenocarcinoma (1)					
Total	54		40	0.74 (0.61, 0.84)			

*Abbreviations*: *N* number of cases; *95 % CI* 95 % confidence interval, *PPV* positive predictive value, *NPV* negative predictive value

The overall performance of the 92-gene classifier for ampullary subtyping, based on concordance of the main cancer type prediction with the histological subtype, was 74 % [95 % CI, 61–84]. For the pancreaticobiliary histological subtype, the sensitivity and specificity of the 92-gene classifier were 85 % [95 % CI, 66–94] and 68 % [95 % CI, 48–84], respectively. For the intestinal histological subtype, the sensitivity and specificity of the 92-gene classifier were 64 % [95 % CI, 46–79] and 88 % [95 % CI, 70–98], respectively (Table 2).

### Histomolecular phenotyping

The histomolecular (morphology plus IHC) pancreaticobiliary phenotype comprises ampullary adenocarcinomas that are of pancreaticobiliary histology with negative CDX2 and positive MUC1 expression by IHC. In contrast, the histomolecular non-pancreaticobiliary phenotype includes ampullary adenocarcinomas that do not exhibit these specific features [3]. Applying this histomolecular criteria results in 14 cases that were classified as pancreaticobiliary phenotype (26 %), and 40 cases that were classified as non-pancreaticobiliary phenotype (74 %) (Table 1 and Table 3). Table 3 shows the comparative data on CDX2 and MUC1 immunostaining across the three classification approaches.

**Table 3:** Differential expression of CDX2 and MUC1 in ampullary adenocarcinomas

	Histology		Histomolecular		92-gene classifier	
	Int	Pb	Non-Pb	Pb	Int	Pb
CDX2+/MUC1-	9	5	14	0	11	3
CDX2-/MUC1+	10	18	14	14	4^a^	23
CDX2+/MUC1+	6	1	7	0	4^b^	2
CDX2-/MUC1-	3	2	5	0	2	3
Total	28	26	40	14	21	31

*Abbreviations*: *Int* intestinal, *Pb* pancreaticobiliary, *Non-Pb* non-pancreaticobiliary

^a^Does not include the single lung adenocarcinoma prediction

^b^Does not include the single gastroesophageal adenocarcinoma prediction

### Comparative prognostic performance

**Fig. 2:**
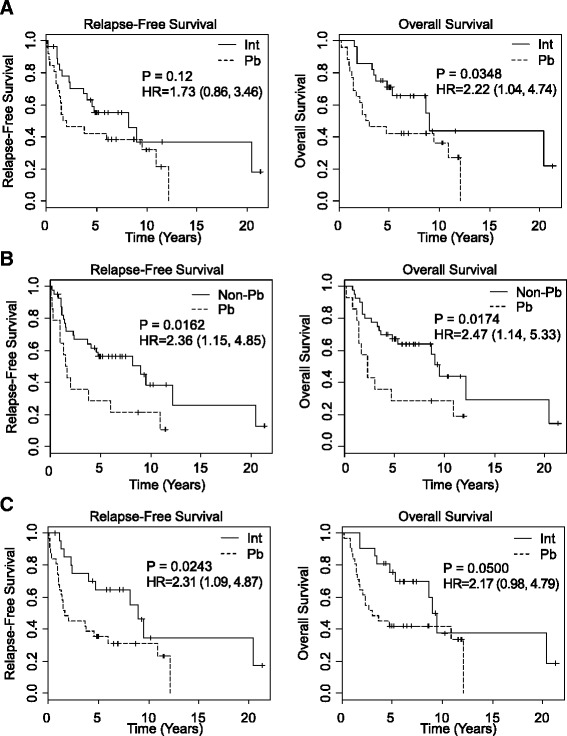
Survival analysis of ampullary adenocarcinoma subtypes stratified by different methods. Kaplan-Meier curves for relapse-free survival and overall survival as a function of (**a**) histological subtype, (**b**) histomolecular phenotype, and (**c**) 92-gene classifier. The solid line represents the intestinal (Int) or non-pancreaticobiliary (Non-Pb) subtypes. The dashed line represents the pancreaticobiliary subtype. For the 92-gene classifier, the single gastroesophageal and single lung adenocarcinoma predictions were excluded

Clinicopathological variables associated with improved OS based on univariate analysis included absence of lymph node metastasis [median OS 113.8 vs 41.8 m, *P* = 0.0343] and negative margins (108.1 vs 18.8 m, *P* = 0.006). Individual immunohistochemical markers were not associated with improved overall survival (Table 1). Figure 2 shows the Kaplan–Meier curves comparing the ability of the three ampullary subtyping methods (histology (morphology alone), histomolecular (morphology plus IHC), and 92-gene classifier) to stratify patients for overall survival and relapse-free survival. The intestinal histological subtype was associated with improved OS compared with the pancreaticobiliary histological subtype (108.1 v 34.6 months; *P* = 0.0348) (Fig. 2a). Histological subtyping did not significantly stratify the cohort for relapse-free survival (RFS) (*P* = 0.12). Histomolecular phenotype, which combines morphological features with CDX2 and MUC1 immunostaining, demonstrated improved OS (median OS 113.8 v 28.0 months; *P* = 0.0174) and RFS (*P* = 0.0162) for the non-pancreaticobiliary phenotype compared to the pancreaticobiliary phenotype (Fig. 2b). For the 92-gene classifier, the difference in OS for the intestine versus pancreaticobiliary main cancer type predictions bordered on significance (median OS 108.1 v 36.4 months; *P* = 0.05) and RFS (*P* = 0.024) (Table 1 and Fig. 2c). After adjusting for nodal and margin status, the three ampullary classifiers did not demonstrate a significant difference in survival for the pancreaticobiliary versus intestinal (or non-pancreaticobiliary) subtype (Additional file 1: Table S1). No statistically significant difference in the prognostic performance was observed across the 3 classifiers (data not shown).

As poor differentiation poses challenges for classification methods that rely on morphological features, we compared the prognostic utility of histological, histomolecular, and the 92-gene classifier in a small subset of high grade or poorly differentiated ampullary tumors (*N* = 24). In this subset the 92-gene classifier demonstrated improved prognosis for intestine compared to pancreaticobiliary main cancer type prediction (OS, *P* = 0.045; RFS *P* = 0.014), whereas no significance was seen with histological based subtyping (OS, *P* = 0.27; RFS *P* = 0.78), or histomolecular phenotype (OS, *P* = 0.31; RFS *P* = 0.14).

### Exploratory analysis of gene subsets associated with survival

**Fig. 3:**
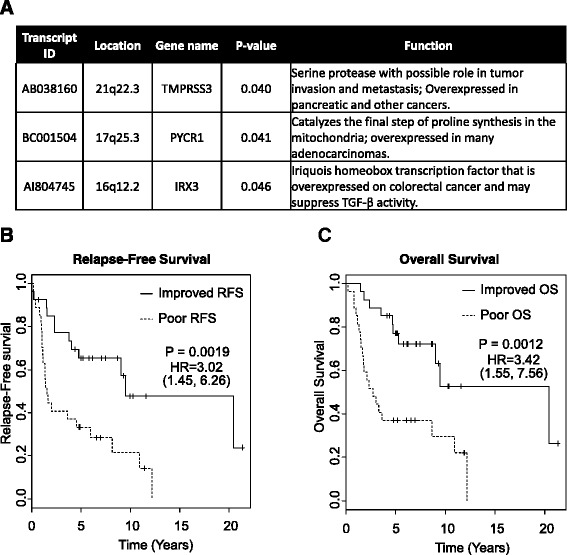
Survival analysis of ampullary adenocarcinoma stratified by the 3-gene model. **a** Location and function of the 3-genes with a significant (*p* < 0.05) association with overall survival. Kaplan-Meier curves for relapse-free (**b**) and overall survival (**c**). The solid line represents tumors with a collective gene expression score less than 0.45 that represent the ampullary subset with better OS. The dashed line represents tumors with a collective gene expression score greater than 0.45 that represent the ampullary subset with worse OS

The outcomes data associated with this cohort of ampullary tumors allow for exploratory analysis of genes whose expression is associated with overall survival, rather than histological subtype. Cox analysis using the gene expression data from the 92-gene classifier identified three genes, IRX3, PYCR1, and TMPRSS3, that showed a significant association (p-value less than 0.05) with overall survival (OS) and relapse-free survival (RFS) (Fig. 3a). The linear combination of gene expression levels from IRX3, PYCR1, and TMPRS33 was used to predict RFS and OS in the 54 patient cohort. As shown in Fig. 3, a 3-gene expression score < 0.45 was associated with improved OS (median OS 74.0 v 32.6 months; HR, 3.42; 95 % CI, 1.55 to 7.56; *P* = 0.0012) and improved RFS (*P* = 0.0019, Fig. 3c). The OS survival group with improved prognosis based on the 3-gene model is comprised mostly of tumors with an intestinal histological subtype (70 %), whereas samples contained within the poor survival group are mostly of the pancreaticobiliary histological subtype (67 %) (Additional file 2: Table S2). The 3-gene linear combination model had an AUC of 0.78 (95 % CI, 0.66 to 0.91) that is similar to the AUC = 0.76 (95 % CI, 0.63 to 0.89) for the 92-gene classifier (Additional file 3: Figure S1).

## Discussion

Identification of patient subgroups based on clinicopathological and molecular characteristics is a validated approach for both tumor subclassification [17] and for the optimal use of targeted therapies [18–23]. The ability to stratify patients into different prognostic and/or treatment groups is particularly important in cancers associated with a diverse range of outcomes such as ampullary adenocarcinomas, which have classically been separated into intestinal or pancreaticobiliary subtypes based on histomorphological assessment [3, 4, 24, 25]. In this blinded study, we demonstrate that a 92-gene cancer classifier showed favorable performance in the classification of ampullary adenocarcinomas into intestinal and pancreaticobiliary histological subtypes, and that these subtypes are prognostically distinct with discrete survival outcomes. The 92-gene cancer classifier represents a non-subjective classification method that demonstrated comparative prognostic performance to histological subtyping (morphology alone) and histomolecular phenotyping (morphology plus IHC).

Demonstration that the 92-gene assay classified ampullary tumors into two statistically significant prognostic groups is a novel finding of this study. Although this work did not demonstrate a single method to have significantly improved ability to prognostically stratify ampullary adenocarcinoma, the comparable prognostic utility of the 92-gene classier with histological subtyping and histomolecular phenotyping suggests that the prognostic capability is partly consistent with histological subtyping. Intriguingly the 92-gene classifier demonstrated an improved ability to identify prognostic subgroups within poorly differentiated ampullary adenocarcinomas, though the sample size for this analysis was small.

Currently, the classification of ampullary adenocarcinomas is performed by non-standardized examination of tumor histomorphology. However, given the subjective nature of histological classification it remains uncertain which methodological approach to ampullary stratification best reflects biology. This variation is highlighted by the substantial differences in histological subtype designations that were observed amongst the three classification methods. Whereas histology alone designated 48 % of the cohort as pancreaticobiliary, histomolecular phenotyping designated 26 % and the 92-gene classifier designated 36 % of the cohort as pancreaticobiliary. Though histomolecular phenotyping also considers differential protein marker expression, this method fundamentally relies upon histomorphological examination. In addition, the challenges of immunohistochemical staining interpretation and the establishment of optimal cut-points for both CDX2 and MUC1 immune reactivity are limitations for the application of histomolecular phenotyping [3, 26]. The 92-gene classifier exhibited sensitivities of 64 % (0.46–0.79) and 85 % (0.66–0.94), and specificities of 88 % (0.70–0.98) and 68 % (0.48–0.84) for the intestinal and pancreaticobiliary histological subtypes, respectively. The differential sensitivities and specificities for the 92-gene assay reflect the increased number of false positive pancreaticobiliary predictions (*N* = 9) within the ampullary-intestine histological subtype in contrast to the false positive intestine predictions (*N* = 3) within the ampullary-pancreaticobiliary histological subtype. Unlike the setting in which the primary cancer site is used as the clinical truth, the use of morphological subtype as the gold-standard reference may be limited by subjective determination.

The performance of the 92-gene classifier is promising for this off-panel tumor, particularly given that the reference training database did not include ampullary adenocarcinoma cases. The observed performance of the 92-gene classifier to subtype these ampullary tumors can be attributed to the discovery methodology of the classifier, which included a data-dependent search for gene combinations from whole genome expression profiling and a genetic algorithm that allowed the gene panel to evolve as a combination [27]. While reduced performance in off-panel tumor types such as ampullary adenocarcinoma is a potential limitation of gene expression based classifiers, these findings support the inherent scalability of the 92-gene assay to recognize a diverse range of tumor types. These data are generally consistent with the utilization of a dichotomous subclassification for ampullary adenocarcinomas. This finding is in agreement with a previous microarray analysis [25] and provides information on cellular context, which points to distinct epithelial origins of ampullary tumors with different outcomes.

Limitations of this study are that, in contrast to the recent histomolecular phenotyping by Chang et al. [3], this current study involves a smaller cohort of patients, likely contributing to the lack of significance for the 3 classifiers in multivariate analysis (Additional file 1: Table S1). Additional limitations of this study were its retrospective nature and use of both untreated and neoadjuvantly treated primary tumors. Further validation of these findings in a larger dataset is needed.

Based on the ability of the 92-gene classifier to measure gene expression in biologically-relevant pathways such as lineage commitment and signal transduction, an exploratory analysis was performed using the cases from this study as a training set to identify a 3-gene expression model that stratified this ampullary cohort into two statistically significant survival groups (HR = 3.42, *P =* 0.0012). Although a majority of cases in the poor survival group had a pancreaticobiliary histological subtype, a significant portion (33 %) of cases in this group had an intestinal histological subtype. Similarly, 30 % of the cases in the improved survival group had a pancreaticobiliary histological subtype, whereas the majority was intestinal (Additional file 2: Table S2). This 3-gene expression model suggests that the collective expression of IRX3, PYCR1, and TMPRSS3 reflects the additional biological importance of tumor invasion, intestinal differentiation, and cellular metabolism [28–34], in the prognosis of ampullary cancers. The increased expression of IRX3 and TMPRSS3 in the poor prognosis ampullary subset is consistent with the biological functions of these genes in promoting tumorigenesis and tumor invasion, respectively [29, 32]. The exploratory 3-gene signature represents a hypothesis-generating findings and further validation is needed.

At the present time the main clinical utility from stratifying ampullary adenocarcinomas results from improved prognostication. The clinical impact of classifying ampullary tumors to inform therapeutic decisions in the adjuvant or metastatic settings has not been proven. Histological subtyping of some ampullary cases in the European Study Group for Pancreatic Cancer (ESPAC)-3 prospective trial demonstrated improved DFS (*P* = 0.01), but not OS (*P* = 0.28). However, due to small number of cases with known histological subtype the outcomes stratified by chemotherapy type and histological subtype were not analyzed [35, 36]. Based upon the reproducible identification of not only distinct prognostic but also biologically unique ampullary subgroups, a clinical trial utilizing subtype guided therapy [e.g. fluoropyrimidine/oxalipliatin for the intestinal ampullary subtype and gemcitabine/cisplatin for pancreaticobiliary ampullary subtype has merit. Additionally, the role of novel approved agents and biomarkers with therapeutic intent needs to be explored in ampullary cancers (e.g. her-2 and RAS for intestinal subtype and IDH1, ARID-1, FGFR for biliary subtypes). One can envision that progress made in pancreas, intestinal and cholangiocarcinomas may directly impact subtypes of ampullary cancers.

## Conclusion

In conclusion, the results of this study demonstrate that the 92-gene assay offers a standardized and reproducible approach beyond the review of morphology and immunohistochemistry to classify ampullary cancers [5, 8]. The 92-gene assay may potentially provide an improved method to classify the molecular subtype of unresectable periampullary cancers in which primary resection tissue is not present and thus the anatomic source of the cancer (pancreatic vs. biliary vs. duodenal vs. ampullary) cannot be determined. A potential next step in the clinical utility of the 92-gene assay is to integrate its use into the broader diagnostic armamentarium of periampullary cancers and allow the molecular subtype designation to guide tailored therapy.

## Additional files

Additional file 1: Table S1. Multivariate analysis of clinicopathological features and the three ampullary subtyping methods. Abbreviations: HR, hazard ratio; CI, confidence interval; Int, Intestinal; Pb, Pancreaticobiliary; Non-Pb, Non-pancreaticobiliary. (PPTX 141 kb) Additional file 2: Table S2. Clinicopathological features of ampullary adenocarcinomas with histologies that are different from their survival groups in the 3-gene model. Abbreviations: OS, overall survival. ^1^T stage, N = 53, one case was carcinoma in situ; ^2^Positive CDX2 expression was defined as modified H score > 35; ^3^Positive MUC1 expression was defined as any positive staining. (DOCX 30 kb) Additional file 3: Figure S1. AUC analysis of the 92-gene classifier and the 3-gene signature in the ampullary cohort. Area under the curve (AUC) analysis for the 92-gene classifier (A) and the 3-gene model (B). (PPTX 76 kb)

## Abbreviations

AUC: Area Under the Curve
FFPE: Formalin-fixed Paraffin Embedded
H&E: Hematoxylin and Eosin
IHC: Immunohistochemistry
Int: Intestine
NPV: Negative Predictive Value
OS: Overall Survival
Pb: Pancreaticobiliary
PPV: Positive Predictive Value
RFS: Relapse-free Survival
ROC: Receiver Operating Characteristic
