# Supplementary material for: Performance and prognostic utility of the 92-gene assay in the molecular subclassification of ampullary adenocarcinoma
Source: BMC Cancer. 2016 Aug 22;16(1):668. doi: 10.1186/s12885-016-2677-3 (PMC4994309; doi:10.1186/s12885-016-2677-3)
Supplement: Additional file 1: Table S1. — Multivariate analysis of clinicopathological features and the three ampullary subtyping methods. Abbreviations: HR, hazard ratio; CI, confidence interval; Int, Intestinal; Pb, Pancreaticobiliary; Non-Pb, Non-pancreaticobiliary. (PPTX 141 kb) [file 12885_2016_2677_MOESM1_ESM.pptx]

## Slide 1
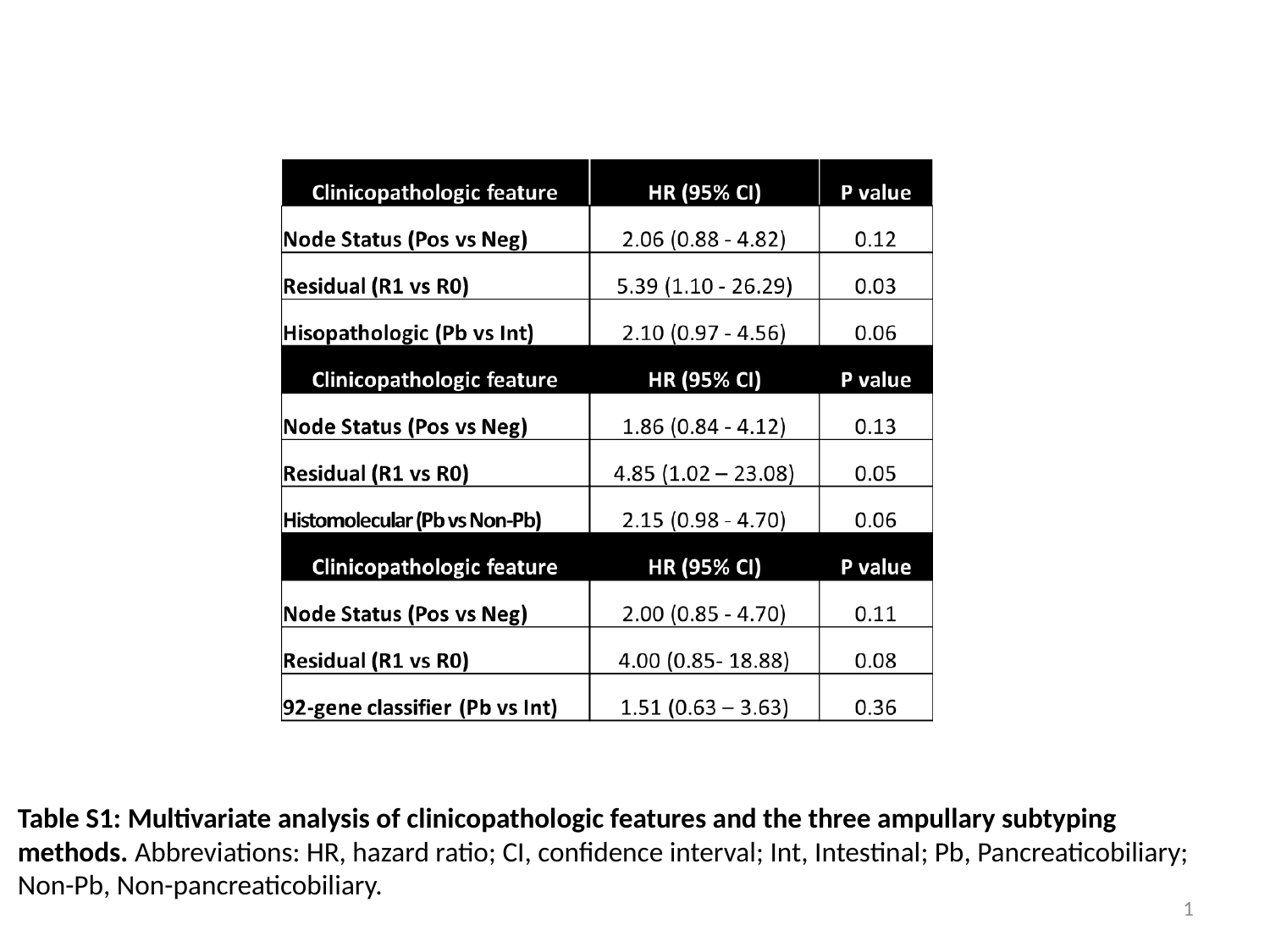

Table S1: Multivariate analysis of clinicopathologic features and the three ampullary subtyping methods. Abbreviations: HR, hazard ratio; CI, confidence interval; Int, Intestinal; Pb, Pancreaticobiliary; Non-Pb, Non-pancreaticobiliary.
1
