# Supplementary material for: Performance and prognostic utility of the 92-gene assay in the molecular subclassification of ampullary adenocarcinoma
Source: BMC Cancer. 2016 Aug 22;16(1):668. doi: 10.1186/s12885-016-2677-3 (PMC4994309; doi:10.1186/s12885-016-2677-3)
Supplement: Additional file 2: Table S2. — Clinicopathological features of ampullary adenocarcinomas with histologies that are different from their survival groups in the 3-gene model. Abbreviations: OS, overall survival. 1T stage, N = 53, one case was carcinoma in situ; 2Positive CDX2 expression was defined as modified H score > 35; 3Positive MUC1 expression was defined as any positive staining. (DOCX 30 kb) [file 12885_2016_2677_MOESM2_ESM.docx]

|  | **Pb histology in the Improved OS group for the 3-gene model (N=8)** | **Int histology in the Poor OS group for the 3-gene model (N=9)** |
| --- | --- | --- |
| **Clinicopathologic feature** | | |
| Male | 5 | 8 |
| Female | 3 | 1 |
| **Age (range in years)** | | |
|  | 40 to 73 | 56 to 73 |
| **T stage^1^** | | |
| T1 | 0 | 0 |
| T2 | 4 | 4 |
| T3 | 3 | 4 |
| T4 | 1 | 1 |
| **N stage** | | |
| N0 | 3 | 3 |
| N1 | 5 | 6 |
| **Grade** | | |
| I | 1 | 0 |
| II | 2 | 7 |
| III | 5 | 2 |
| **Mucinous** | | |
| Negative | 8 | 9 |
| Positive + signet ring | 0 | 0 |
| **Perineural invasion** | | |
| Negative | 7 | 7 |
| Positive | 1 | 2 |
| **Lymphovascular invasion** | | |
| Negative | 7 | 6 |
| Positive | 1 | 3 |
| **Perioperative treatment** | | |
| Neoadjuvant +/or adjuvant | 4 | 6 |
| None | 4 | 3 |
| **Adenoma precursor lesion** | | |
| Absent | 8 | 3 |
| Present | 0 | 6 |

**Table S2**

|  |  |  | |
| --- | --- | --- | --- |
| **Supplementary Table 2 continued** | **Pb histology in the Improved OS group for the 3-gene model (N=8)** | | **Int histology in the Poor OS group for the 3-gene model (N=9)** |
| **Clinicopathologic feature** | | | |
| **Resection margin** | | | |
| R0 | 8 | 8 | |
| R1 | 0 | 1 | |
| **CK7 expression** | | | |
| Negative | 6 | 2 | |
| Positive | 2 | 7 | |
| **CK20 expression** | | | |
| Negative | 7 | 6 | |
| Positive | 1 | 3 | |
| **CDX2 expression^2^** | | | |
| Negative | 5 | 4 | |
| Positive | 3 | 4 | |
| **MUC1 expression^3^** | | | |
| Negative | 3 | 5 | |
| Positive | 5 | 4 | |
| **Mixed histology** | 2 | 4 | |
| **Histomolecular Phenotype** | | | |
| Non-Pancreaticobiliary | 4 | 9 | |
| Pancreaticobiliary | 4 | 0 | |
| **92-gene Classifier** | | | |
| Intestinal | 3 | 3 | |
| Pancreaticobiliary | 4 | 6 | |
| Lung adenocarcinoma | 1 | 0 | |
| **Supplementary Table 2: Clinicopathologic features of ampullary adenocarcinomas with histologies that are different from their survival groups in the 3-gene model.** Abbreviations: OS, overall survival. ^1^ T stage, N = 53, one case was carcinoma in situ; ^2^ Positive CDX2 expression was defined as modified H score > 35;  ^3^ Positive MUC1 expression was defined as any positive staining. | | | |
