# Supplementary material for: Performance and prognostic utility of the 92-gene assay in the molecular subclassification of ampullary adenocarcinoma
Source: BMC Cancer. 2016 Aug 22;16(1):668. doi: 10.1186/s12885-016-2677-3 (PMC4994309; doi:10.1186/s12885-016-2677-3)
Supplement: Additional file 3: Figure S1. — AUC analysis of the 92-gene classifier and the 3-gene signature in the ampullary cohort. Area under the curve (AUC) analysis for the 92-gene classifier (A) and the 3-gene model (B). (PPTX 76 kb) [file 12885_2016_2677_MOESM3_ESM.pptx]

## Slide 1
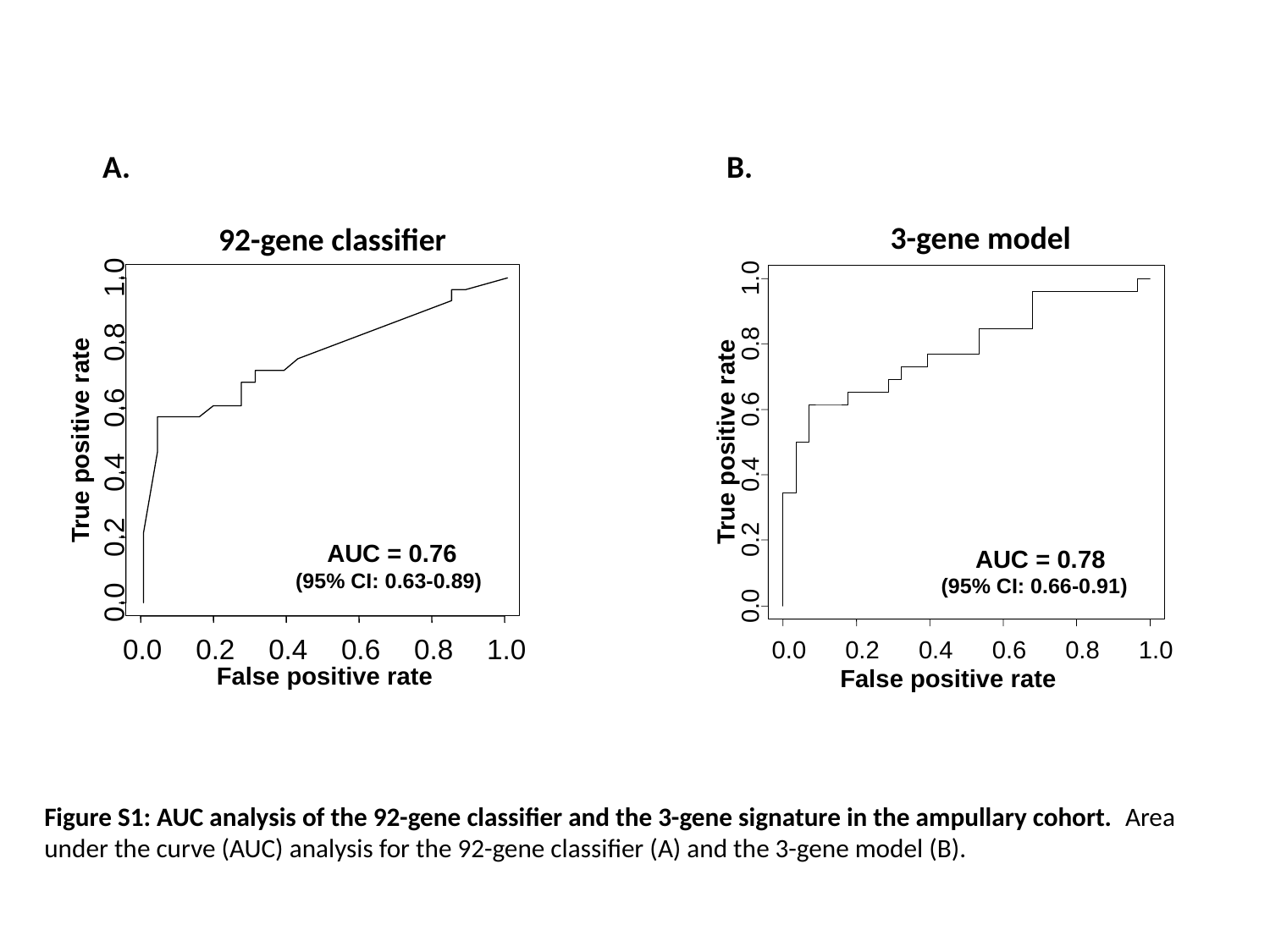

A.
B.
1.0
0.8
0.6
True positive rate
0.4
0.2
0.0
0.0
0.2
0.4
0.6
0.8
1.0
False positive rate
AUC = 0.76
(95% CI: 0.63-0.89)
1.0
0.8
0.6
True positive rate
0.4
0.2
AUC = 0.78
(95% CI: 0.66-0.91)
0.0
0.0
0.2
0.4
0.6
0.8
1.0
False positive rate
3-gene model
92-gene classifier
Figure S1: AUC analysis of the 92-gene classifier and the 3-gene signature in the ampullary cohort. Area under the curve (AUC) analysis for the 92-gene classifier (A) and the 3-gene model (B).
